# A dual frailty model for lifetime analysis in maritime transportation

**DOI:** 10.1007/s10985-019-09463-3

**Published:** 2019-02-19

**Authors:** Robin Henderson, Ralitsa Mihaylova, Paul Oman

**Affiliations:** 1 School of Mathematics, Statistics and Physics, Newcastle University, Newcastle, UK; 2 School of Engineering, Newcastle University, Newcastle, UK; 3 Department of Mathematics, Physics and Electrical Engineering, Northumbria University, Newcastle, UK

**Keywords:** Bayes, Clarksea index, Ownership duration, Partial likelihood, Proportional intensity, Random effects, Sentiment

## Abstract

We consider changes in ownership of commercial shipping vessels from an event history perspective. Each change in ownership can be influenced by the properties of the vessel itself, its age and history to date, the characteristics of both the seller and the buyer, and time-varying market conditions. Similar factors can affect the process of deciding when to scrap the vessel as no longer being economically viable. We consider a multi-state approach in which states are defined by the owning companies, a sale marks a transition, and scrapping of the vessel corresponds to moving to an absorbing state. We propose a dual frailty model that attempts to capture unexplained heterogeneity in the data, with one frailty term for the seller and one for the buyer. We describe a Monte Carlo Markov chain estimation procedure and verify its accuracy through simulations. We investigate the consequences of mistakenly ignoring frailty in these circumstances. We compare results with and without the inclusion of frailty.

**Electronic supplementary material:**

The online version of this article (10.1007/s10985-019-09463-3) contains supplementary material, which is available to authorized users.

## Introduction

We consider an event history approach to the analysis of ownership duration data, with focus on maritime transport. A change of ownership of an item—in our case an ocean-going vessel—can be considered as an event in time with the interesting feature that each event has a dual interpretation: as a *sale* by one company and a *purchase* by another. We will explore and apply methods for the analysis of such data in this paper.

Examples of event history analyses of ownership duration to date include studies of the housing market (Archer et al. 2010); corporate ownership and equity duration (Bøhren et al. 2005); foreign owners and plant survival for companies (Kronborg and Thomsen 2009) and, in the transport sector, automotive vehicle ownership (Iwata and Matsumoto 2016). The use of event history analysis in the maritime related literature, however, is very limited, though some notable examples include work on survival of Norwegian shipping companies (Tenold and Aarbu 2011) and an investigation into the effectiveness of ship inspections (Bijwaard and Knapp 2009). It is somewhat surprising that research on length and pattern of ownership in shipping is so scarce (Stott 2014), given that the majority of world trade is transported by sea (UNCTAD 2017). We, therefore, suggest that an examination of how event history analysis could be applied in this field is long overdue and that any such analysis should accommodate the dual interpretation of an event, as explained in the first paragraph.

A first approach to such an event history analysis of vessel ownership is usually to examine covariates in an attempt to explain the variation in the time taken until the specified event occurs. Such times are defined as the length of time from some start event (when the vessel is bought) until the time to some stop event (when the vessel is sold or scrapped) but with ownership times being censored for those vessels that are owned by a company at the end of the observation period.

**Fig. 1:**
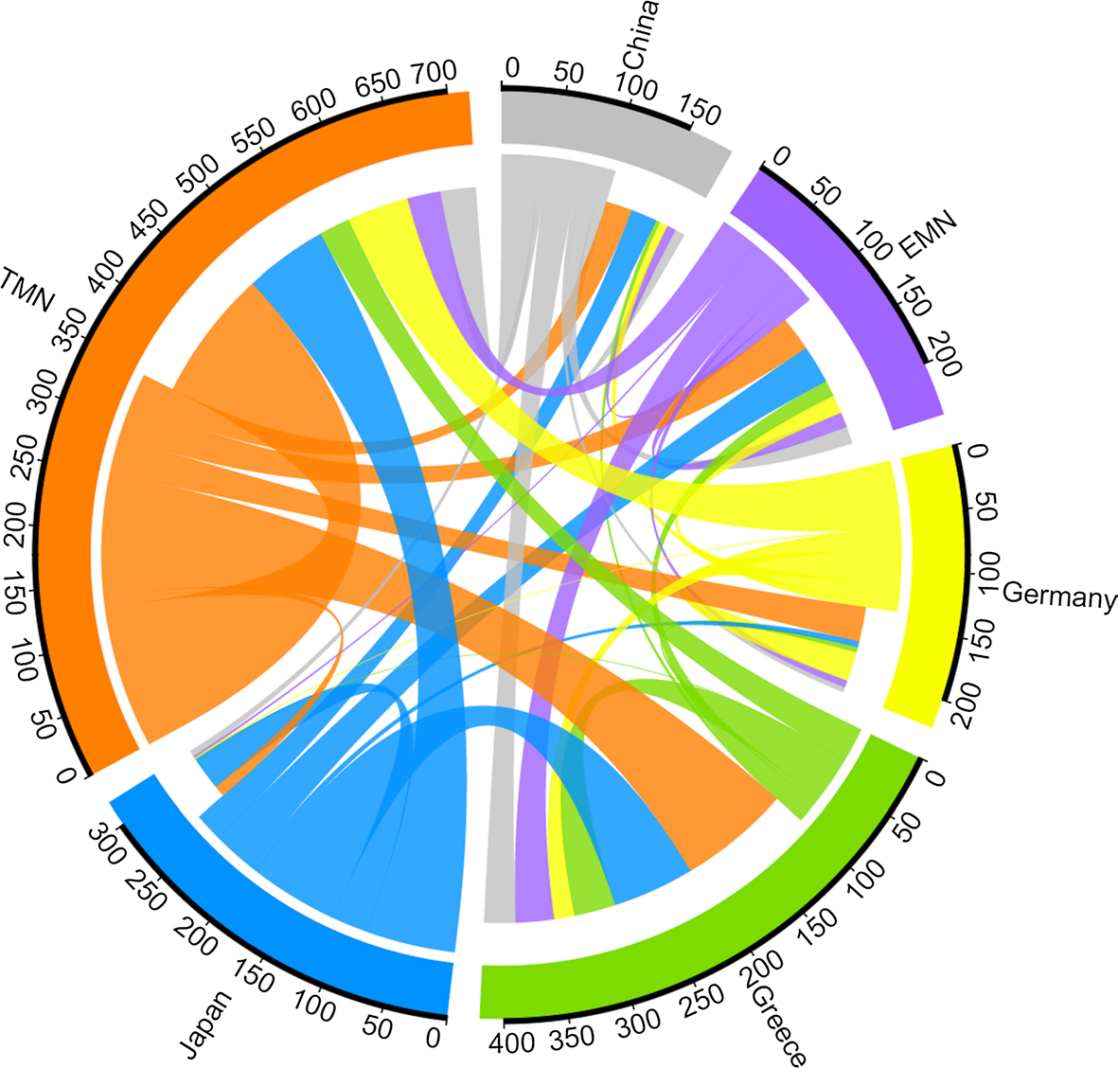
First changes in ownership, classified by state-group of selling and buying companies. The start and end width of each line is proportional to the number of vessels. The outermost circle represents the number of transactions involving companies in the state-group. The next circle represents sales and the third represents purchases. The plot was produced using methodology developed by Sander et al. (2014). TMN is traditional maritime nations and EMN is emerging maritime nations

One drawback is that not all possible reasons for the variation in the intensity function may have been included in the model as there are many factors that could affect the length of ownership of a vessel (Stopford 2009). In Fig. 1, for example, we show a transition diagram for sales between the first and second owners of the vessels in our data set. The diagram will be explained and discussed further in the next section but it is clear that the relationship between buyers and sellers is complex and there is potential for significant heterogeneity in behaviour.

We can model such heterogeneity with *random effects*, an approach that was first introduced by Beard (1959) with the alternative term *frailty* coined by Vaupel et al. (1979) 20 years later. Much work on frailty in a variety of settings soon followed, particularly important examples being Oakes (1982) and Hougaard (1986). This growing body of work was enhanced when Aalen demonstrated clearly how heterogeneity could be modelled (Aalen 1987), while warning that ignoring frailty could have a significant impact on estimating effects. In a series of further papers Aalen went on to provide the theoretical underpinning for modelling the population hazard for the univariate frailty model, making the inclusion of frailty more accessible and routine (Aalen 1988, 1992, 1994; Aalen and Tretli 1999; Aalen et al. 2014).

It is recommended that an attempt should always be made to include frailty if it is possible that heterogeneity is present. We therefore look to include sources of possible unexplained variation in our current shipping scenario in a novel approach that contrasts the frailty values of buyers and sellers. More explicitly, one possible source of heterogeneity involves the companies who own the vessels as they may all be susceptible to sales at different rates. In addition, companies looking to buy a vessel may also influence the rate of sales as each purchase also needs a willing buyer and all companies are willing to varying extents. It, therefore, seems sensible to combine in the intensity the willingness of companies to sell with the willingness of other companies to buy. This can be partially accommodated by including covariates for the vessel, the company that owns the vessel and the company that wants to buy the vessel. We cannot, however, be confident that we have captured all of the effects that may influence the sale of a vessel so we look to include separate frailty terms for both the selling company and the buying company, an approach we call *dual frailty* and explain as follows.

Let us first define ‘sentiment’ *S* as a measure of whether a company is looking to sell a vessel or buy a vessel. We shall assume that the intensity function for a sale is in the familiar Cox proportional intensity form with our sentiment value influencing the intensity from within the exponential term (a full expression for the intensity will be given in Sect. 3). We assume that positive values of *S* are assigned to companies willing to sell (thereby increasing the intensity function for a sale) and negative values assigned to companies willing to buy (thereby decreasing the intensity for a sale). For mathematical expedience, we choose to amend the form of the intensity with the frailty term $Z=\exp (S)$ so that it appears outside of the exponential and we can assign it the familiar gamma distribution to aid our analysis.

After describing the data and problem in Sect. 2, we will develop our model and estimation procedure in Sect. 3. We shall then explore with simulations in Sect. 4, report our results in Sect. 5 and discuss possible extensions in Sect. 6.

## Transactions data

The ownership structure in shipping is fragmented, with various different definitions, the two most important of which are *registered* and *beneficial* owner respectively (Veenstra and Bergantino 2000). The registered owner of a vessel is the legal title appearing on the vessel’s registration but this often changes in shipping for various reasons, including tax and liability. The beneficial owner, also referred to as ultimate owner (Kang and Kim 2012), is the entity that gains “the ultimate financial benefit from a vessel’s operation” (Fox 2005).

We consider the beneficial ownership records of 1999 commercial vessels built between 1987 and 2007 and followed until 2015. The data were collected by Ralitsa Mihaylova as part of her Ph.D. programme, and were obtained via individual inspection of records made available by two leading shipping data providers: IHS Maritime and Trade’s online tool Sea-web and Clarksons Research Services Ltd (CRSL). Mihaylova (2018) provides full information.

Table 1 gives an overview of ownership and sales or scrap events for each vessel category. The entry point for a vessel is the date of the delivery to the first owner, which is the date the vessel enters into operation (Stott 2014). Vessels were followed until 2015 unless they were scrapped earlier. The shortest recorded lifetime was for a vessel that was scrapped at 13.5 years, and the longest was for a vessel that was still in use after 28.75 years. Working lifetimes were typically in the region of 25 years: Kaplan–Meier estimates are 80% still in use at 20 years but only 6% in use at 28.75 years. The maximum number of owners recorded in the data was six, with 47% of vessels not being sold at all during the follow-up period.

**Table 1:** Transactions data summary

Type	Number				Total owners					
	Vessels	Censored	Scrapped	Sales	1	2	3	4	5	6
Dry bulk	797	624	173	883	289	258	156	68	16	10
Tanker	585	468	117	450	284	191	77	27	6	0
Container	617	481	136	337	372	171	59	12	3	0
All	1999	1573	426	1670	936	620	292	107	25	10

Mihaylova (2018) analysed ownership durations for these data, using standard survival analysis techniques rather than the dual frailty and multistate approach considered here, and without allowance for buyer effects. Our selection of covariates is based on the findings there.

We used four vessel-level covariates: type of vessel, deadweight (scaled to a multiple of 10,000 tonnes), speed (knots) and number of previous owners, the latter being time-varying. The vessel types are dry-bulk vessels, tankers and containers, but as dry-bulk vessels and tankers both represent the bulk market (dry and liquid respectively) in our analyses we simply use a binary indicator for container or otherwise. The deadweight reflects the cargo-carrying capacity of a vessel.

The number of companies involved in the commercial history of the vessels was 1125. Not all were active at the same time. We identified 413 companies as being active at the beginning of the follow-up period. The number rose steadily to a stable level around 860 from 2005 to 2008, and then, following the financial crash, declined to 696 at the end of follow-up. We classified each company by type and nationality. We took four company types (private, public, financial and state-owned) and six nationality categories (China, Germany, Greece, Japan, other traditional maritime nations and emerging maritime nations). Nationality is associated with beneficial ownership as it reflects the country where the primary economic contribution ends up, which may or not be where the company is registered or where the owner is based. The data on nationality was retrieved from Sea-web. The categories “traditional maritime nations” (TMN) and “emerging maritime nations” (EMN) are based on the maritime traditions framework developed by Alderton and Winchester (2002). As company-level covariates we defined appropriate indicator variables for the classification, with baseline taken to be a privately-owned company associated with a traditional maritime nation.

There are many potential exogenous variables, as discussed in Mihaylova (2018). For simplicity we use just one, the logged Clarksea index, which is a good proxy for market conditions. It is a weighted average of the daily earnings of the main vessel types, where the weighting is based on the number of vessels in each fleet segment provided by CRSL. We chose the Clarksea index because freight earnings are believed to trigger activities within the shipping market, including the ordering and sale and purchase of vessels (Abouarghoub et al. 2012). In the analyses reported in Sect. 5 we used the Clarksea index at the calendar time of potential sales, and also lagged by six months. In addition we allowed interaction between the index and vessel type.

Most of the covariates are categorical: their frequencies are given in Table 2. There are four quantitative covariates: deadweight, speed, number of previous owners and the Clarksea index. Deadweight ranged from $1.2 \times 10^4$ tonnes to $44 \times 10^4$ tonnes, with mean $8\times 10^4$ tonnes. Speeds ranged from 12 to 26.5 knots with mean 16.7 knots. Vessels had up to five previous owners in the time period. The logged Clarksea index varied between 8.970 and 10.789: its value over time is given later, in Fig. 4.

**Table 2:** Overview of categorical covariates

Covariate	Levels	Counts
Vessel type	Container	617
	*Bulker/tanker*	1382
Company type	*Private*	895
	Financial	26
	Public	159
	State	45
Company nationality	China	117
	EMN	184
	*TMN*	380
	Germany	101
	Greece	245
	Japan	98

Italics indicate baseline levels

Finally for this section, to illustrate some of the complexity of the transactions data, Fig. 1 represents the transitions from first to second owner based on nationality. The number of vessels participating in the transition from first to second owner is represented by the outermost circle. The outgoing flows show the number of vessels sold (second circle) and therefore the colour of the flow corresponds to the colour of the owner nationality. The incoming flows (third circle) represent the number of vessels that were bought. From the diagram, it is clear that Japanese and German owners are more involved in purchasing new vessels than acquiring second-hand tonnage, whereas Greek owners appear to be more active in the second-hand market.

## Model and estimation

### Preliminaries

The data consists of information on *K* vessels that are delivered in a calendar time interval $(\tau _1, \tau _2)$, with follow-up to time $\tau$. For each vessel we have the delivery date, the dates, if any, at which the vessel is sold from one company to another, and the date of scrapping if appropriate. The final time $\tau$ censors the observed history for vessels not yet scrapped.

There are *N* companies which are potential buyers or sellers of vessels for at least part of the observation interval. Events of interest are the sale of a vessel from one company to another or the scrapping of the vessel by the current owner. In each case the event intensity is expected to depend upon both calendar time *t* and vessel age *a*. Let $t_v(a)$ be the calendar time at which vessel *v* reaches age *a*.

First we consider covariates. These fall into three groups:

- $x_v^V(t)$ is a $p_1$-vector of covariates associated with vessel *v* at calendar time *t*. Some of these may be static, such as deadweight for instance, whilst others may be dynamic summaries of the vessel’s history, such as number of previous owners or duration of current ownership.
- $x_c^C(t)$ is a $p_2$-vector of covariates associated with company *c*. In principle these can vary with calendar time although in our application they are time-fixed characteristics of the company.
- $x^E(t)$ is a $p_3$-vector of exogenous covariates reflecting market conditions or time trends perhaps.

Next we have two at-risk indicators. Since companies may not be active throughout the full observation window, the first indicator $Y^C_c(t)$ is taken to be one if company *c* is known to be active at calendar time *t* and is zero otherwise. The second at-risk indicator $Y^V_v(a)$ is defined to be one if vessel *v* is still in use and hence liable to be sold at age *a*, and is taken to be zero if the vessel has been scrapped or censored.

Further, we associate with each company *c* an unobserved random variable $S_c(t)$ that captures the *sentiment* of the company, with positive values implying a willingness to capitalise on assets and sell vessel holdings, and negative values implying an unwillingness to capitalise and a preference for investment in new vessels. It will be convenient to denote $Z_c(t)=\exp \{S_c(t)\}$. To be consistent with biostatistical terminology we will refer to $Z_c(t)$ as “frailty” and reserve “sentiment” for $S_c(t)$ as convenient. For simplicity, we will assume for our first analyses that the random effects are time-constant, $Z_c=Z_c(t)$ and $S_c=S_c(t)$. We will come back to this issue later.

Finally, let $\mathcal {F}_t$ denote the observed history of events and covariate evolution up to calendar time *t* and let *Z* be the *N*-vector of frailties.

### Dual frailty model

At age *a* a generic vessel *v* may be sold or scrapped. If sold, then any of the companies that are active at the relevant calendar time may be the purchaser. Hence we have a multistate problem and our approach will be based on modelling the transition intensities (e.g., Andersen and Keiding 2002).

We assume the frailty effects $Z_c$ are independent gamma random variables with mean one and variance $\xi$. Conditioning on the combined vector of frailties *Z* and prior history, let $\alpha _{v}(a, b \mid \mathcal {F}_{t_v(a)}, Z)$ be the transition intensity for a sale of vessel *v* to buying company *b* at vessel age *a*, which occurs at calendar time $t=t_v(a)$. We will use $s=s(a,v)$ to indicate the selling company, ie the current owner. We assume a semi-parametric multiplicative model

1 $$\begin{aligned} \alpha _v( a, b \mid \mathcal {F}_{t_v(a)}, Z )= & {} \exp \{ S_s(t)- S_b(t) \} \nonumber \\&\times \,\alpha _{0}(a)\exp \left\{ \beta _V x_v^V(a) +\beta _S x_s^C(t)+\beta _B x_b^C(t)+ \beta _E x^E(t) \right\} \nonumber \\= \,& {} Z_s Z_b^{-1} \alpha _0(a) \exp \left\{ \beta _V x_v^V(a) +\beta _S x_s^C(t)+\beta _B x_b^C(t)+ \beta _E x^E(t) \right\} \nonumber \\= \,& {} Z_s Z_b^{-1} \alpha _0(a) R_v(a, b ; \beta ), \end{aligned}$$

say, where $\beta =(\beta _V, \beta _S, \beta _B, \beta _E)$, $R_v(a, b; \beta )$ is the relative risk determined by covariates and $\alpha _0(a)$ is the baseline intensity. In Eq. (1) the selling company is uniquely determined by the filtration as the current owner of the vessel, but the buyer *b* can be any of the other companies that are currently active at the relevant calendar time. The overall sales intensity for the vessel is thus

2 $$\begin{aligned} \alpha _v(a \mid \mathcal {F}_{t_v(a)}, Z) = \sum _{b \ne s(a,v)} Y^C_b\{t_v(a)\} \alpha _v( a, b \mid \mathcal {F}_{t_v(a)}, Z ), \end{aligned}$$

and the cumulative sales intensity of vessel *v* to age *u* is

3 $$\begin{aligned} A_v(u \mid \mathcal {F}_u, Z) = \int _0^u Y^V_v(a) \alpha _v(a \mid \mathcal {F}_{t_v(a)}, Z) da. \end{aligned}$$

In (1), $\beta _V$ measures how vessel-level characteristics affect the intensity of sales, $\beta _S$ measures how the owning company characteristics influence the likelihood of selling, and $\beta _B$ is similarly used to measure how the characteristics of potential buyers affect the intensity of sales. The final regression coefficient $\beta _E$ is used in an attempt to capture exogenous market conditions or time trends. There are two frailty terms in (1), one for the selling company and one for the buying company. A sale from company *s* to company *b* is more likely, given covariates, when $Z_s$ is high and $Z_b$ is low, and less likely if the circumstances are reversed. We are not aware of any previous work on dual frailty effects of this type.

We make no assumptions about $\alpha _0(a)$. Thus we do not specify the form of the vessel age effect, but within (1) we have fully specified the form of the calendar time effect by allowing time trends in $x^E(t)$. Our reason is that this form allows us to include economic indicators in $x^E(t)$, whose effects would not be identifiable if we had a nonparametric baseline in calendar time *t* rather than age *a*.

Vessels can be scrapped as well as sold. Our model for the corresponding cause-specific hazard is

4 $$\begin{aligned} \lambda _v( a \mid \mathcal {F}_{t_v(a)}, Z )= \,& {} Z_s \lambda _0(a) \exp \left\{ \theta _V x_v^V(a) +\theta _S x_s^C(t) + \theta _E x^E(t) \right\} \nonumber \\=\, & {} Z_s \lambda _0(a) Q_v(a ; \theta ), \end{aligned}$$

say, where $\theta =(\theta _V, \theta _S, \theta _E)$ and $Q_v(a ; \theta )$ is the non-frailty relative risk. There is no buying company effect for scrap, which is of course an absorbing state. We use

5 $$\begin{aligned} \varLambda _v(u \mid \mathcal {F}_u, Z) = \int _0^u Y^V_v(a) \lambda _v(a \mid \mathcal {F}_{t_v(a)}, Z) da \end{aligned}$$

to denote the cumulative scrap hazard of vessel *v* up to age *u*.

### Estimation

Given *Z*, partial likelihood methods are available for estimation of $\beta$ and $\theta$. How best to deal with *Z* is not so obvious however. A variety of methods can be used for standard frailty models for survival data, including, inter alia, EM (e.g., Barker and Henderson 2005), penalised likelihood (e.g., Androukalis et al. 2012), h-likelihood (e.g., Ha et al. 2001), or marginal approaches after integrating out the frailties. None of these lend themselves to our dual frailty problem.

Instead, we will take a Bayesian approach, estimating *Z*, $\beta$ and $\theta$ using Markov chain Monte Carlo and using versions of the Breslow estimator for baselines. We suggest standard Metropolis–Hastings for the regression parameters $\beta$ and $\theta$ and the log of the frailty variance $\xi$, with random walk proposals. For *Z*, a Gibbs sampling approach is more efficient and is feasible given our construction.

To see this, consider the contribution of vessel *v* to the full-data likelihood. In short and loose notation, this is

$$\begin{aligned} L_v = \left( \prod _\mathrm{sales} \alpha _v \right) e^{-A_v} \lambda _v^\delta e^{-\varLambda _v}, \end{aligned}$$

where $\delta$ is an indicator of the vessel being scrapped, $\alpha _v$ and $\lambda _v$ are the sales intensity and scrap hazard, and $A_v$ and $\varLambda _v$ are the cumulative intensities. From (1)–(5) each intensity $\alpha _v$ is proportional to $Z_sZ_b^{-1}$ for some $Z_s$ and $Z_b$ and each $\lambda$ is a multiple of the frailty $Z_c$ of the final owning company. The cumulative intensity $A_v$, given at (3), is the integral of the sales intensity (2). The integral can be broken into disjoint segments, one per ownership period, within each of which the integrand is a sum of terms involving products like $Z_sZ_b^{-1}$ for the selling and potential buying companies. The cumulative scrap intensity $\varLambda _v$ can similarly be broken into disjoint segments, in each of which the integrand is proportional to the frailty of the current owner.

Taken over all vessels, the above means that the full-data likelihood considered as a function of a frailty $Z_c$ for a generic company *c*, is proportional to

$$\begin{aligned} Z_c^M \exp \{-D_1 Z_c -D_2 Z_c^{-1}\}, \end{aligned}$$

for some *M* and non-negative $D_1$ and $D_2$. The value of *M* is simply the difference between the total number of vessels sold or scrapped by company *c* and the total number vessels purchased by that company. The value of $D_1$ depends upon the cumulative sales and scrap intensities for vessels owned by the company, while $D_2$ is the cumulative intensity for purchases. Expressions for $D_1$ and $D_2$ are unwieldy to write down and hence omitted, but they are straightforward to program. Note that $D_1$ and $D_2$ both involve frailty terms for other companies.

When the likelihood is combined with an independent gamma $\varGamma (1/\xi , 1/\xi )$ prior for $Z_c$, we see that the posterior distribution of $Z_c$, given the data, the parameters, and the frailty values for all other companies, is proportional to

$$\begin{aligned} Z_c^{M+1/\xi -1 } \exp \{-(D_1+1/\xi )Z_c -D_2 Z_c^{-1}\}. \end{aligned}$$

This is a generalised inverse gamma distribution (Hougaard 2000, p. 508). The R package GIGrvg includes a routine rgig to simulate from this distribution and hence Gibbs sampling can be implemented.

This leaves the baseline sales intensity and scrap hazard, $\alpha _0(a)$ and $\lambda _0(a)$ respectively. Given our use of MCMC, we take the common approach of profiling out the baseline intensity and hazard functions at each iteration. For fixed frailties *Z* and regression parameters $\beta =(\beta _V, \beta _S, \beta _B, \beta _E)$ and $\theta =(\theta _V, \theta _S, \theta _E)$, versions of the Breslow estimators of the cumulative baseline sales intensity

$$\begin{aligned} A_0(u)= \int _0^u \alpha _0(a) da \end{aligned}$$

and cumulative baseline scrap hazard

$$\begin{aligned} \varLambda _0(u) =\int _0^u \lambda _0(a) da \end{aligned}$$

are available. Letting $dN_\mathrm{sale}(a)$ and $dN_\mathrm{scrap}(a)$ be the total numbers of sales and scraps of vessels at age *a* respectively, the estimators are

6 $$\begin{aligned} {\hat{A}}_0(u ; \beta , Z) = \sum _{a \le u} \frac{ dN_\mathrm{sale}(a) }{ \sum _v \sum _{b \ne s(a,v)} Y^V_v(a) Y^C_b(t_v(a)) Z_{s(a,v)} Z_b^{-1} R_v(a,b; \beta ) } \end{aligned}$$

and

7 $$\begin{aligned} {\hat{\varLambda }}_0(u ; \theta , Z) = \sum _{a \le u} \frac{ dN_\mathrm{scrap}(a) }{ \sum _v Y^V_v(a) Z_{s(a,v)} Q_v(a ; \theta ) } \end{aligned}$$

respectively.

Finally, we note that an alternative hybrid approach is to use partial likelihood for estimation of the regression parameters $\beta$ and $\theta$ at each iteration of an MCMC for $\xi$ and *Z*. The final variance estimate needs to combine the within-chain variation arising through variability in *Z* with the information-based estimate conditional upon *Z*.

## Simulations

To investigate performance of the estimation procedure we undertook a small simulation study. For simplicity we did not consider vessel scrapping, we assumed there were no exogenous covariates $x^E(t)$ and we assumed that all companies were active throughout the study period.

We took $N=500$ companies and $K=1000$ vessels. We assumed two time-constant vessel-level covariates $X^V$. The first was binary and the second uniform on $\pm \,20$, loosely representing vessel category and centred deadweight respectively. We also assumed two time-constant company level covariates $X^C$, in this case both binary. All covariates were independent of the others.

We set the vessel regression parameters to be $\beta _V$ = (0.3, − 0.01), the selling company parameters to be $\beta _S$ = (0.4, − 0.2) and the buying company parameters to be $\beta _B$ = (− 0.2, 0.1). We simulated in discrete time, with baseline intensity $1 \times 10^{-5}$ per month for each vessel and potential buyer combination, and with follow-up censored at 500 months for all vessels. We considered two choices of frailty variance: $\xi$ = 0.2 and $\xi$ = 0.4. These parameter choices typically led to around 1800–2000 transactions in each simulated data set.

We used Metropolis–Hastings for the regression parameters $\beta$ and for log($\xi$), with random walk proposals and zero-mean Gaussian increments. After some trial and error, we selected the standard deviation for the increments to be 0.07 for all regression parameters other than the continuous vessel-level covariate (which is on a different scale), for which we used standard deviation 0.002. For log($\xi$) the increment standard deviation was 0.02. Initial values for the regression parameters were taken from a no-frailty analysis, all frailties $Z_c$ were initially set at one, and the frailty variance $\xi$ was initialised at the low value of 0.01. For each value of $\xi$ we took 1000 replications, each with 3000 iterations for burn-in and a further 10,000 iterations for estimation.

Table 3 summarises our results, for the full MCMC and also for standard proportional intensity analyses in which frailty is mistakenly ignored. In that case there is clear attenuation of the regression parameters for vessels and selling companies, $\beta _V$ and $\beta _S$ respectively, as might be expected when frailty is ignored (Henderson and Oman 1999). Interestingly, there is no evidence of attenuation for the buying-company parameters $\beta _B$. We explore this issue further in supplementary material.

When frailty is properly accounted for our procedure seems to work well, as it should. All means are within simulation noise of the true parameter values, the estimated standard errors closely match the empirical values, and the number of iterations is adequate.

**Table 3:** Simulation results

	True	Ignored frailty			MCMC		
		Mean	Est SE	Emp SE	Mean	Est SE	Emp SE
$$\xi =0.2$$							
$\beta _{V}$	0.300	0.261	0.049	0.050	0.299	0.053	0.052
	− 0.010	− 0.009	0.002	0.002	−0.010	0.002	0.002
$\beta _{S}$	0.300	0.273	0.048	0.070	0.299	0.068	0.066
	0.400	0.357	0.050	0.071	0.401	0.071	0.069
$\beta _{B}$	− 0.200	− 0.201	0.049	0.070	− 0.202	0.070	0.067
	0.100	0.104	0.049	0.072	0.104	0.071	0.068
$\xi$	0.200				0.199	0.020	0.019
$$\xi =0.4$$							
$\beta _{V}$	0.300	0.218	0.050	0.050	0.298	0.057	0.052
	− 0.010	− 0.007	0.002	0.002	− 0.010	0.002	0.002
$\beta _{S}$	0.300	0.253	0.049	0.123	0.304	0.087	0.082
	0.400	0.301	0.051	0.119	0.398	0.089	0.081
$\beta _{B}$	− 0.200	− 0.208	0.050	0.141	− 0.205	0.092	0.088
	0.100	0.109	0.050	0.146	0.104	0.093	0.092
$\xi$	0.400				0.398	0.032	0.032

Estimates are from 1000 simulations, each involving 500 companies and 1000 vessels

Columns 4–6 are from (partial) likelihood analyses, ignoring frailty

Columns 7–9 are from MCMC analyses, properly allowing for frailty

‘Est SE’ is based on the average within-run variance, and ‘Emp SE’ is based on the observed variance of mean values across replications

## Application to vessel trading

**Fig. 2:**
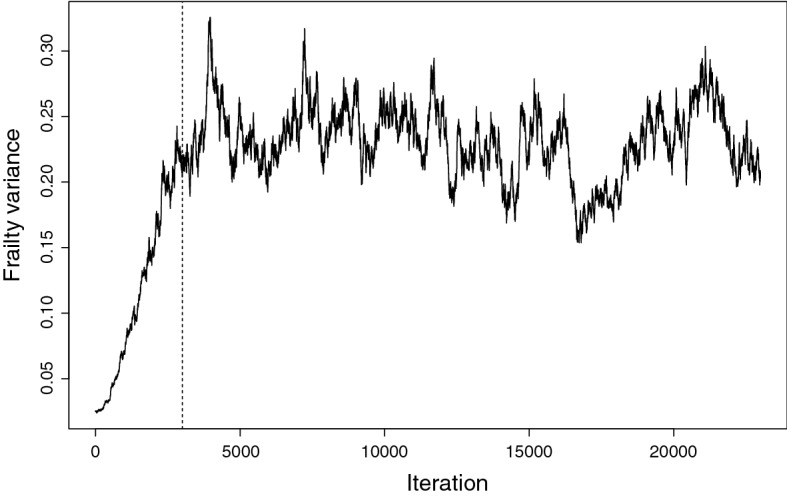
MCMC frailty variance trace plot. Values to the left of the vertical line at iteration 3000 were discarded as burn-in

Maximised log partial likelihoods under a no-frailty model are in Table 4 and coefficient estimates with and without frailty are in Tables 5, 6 and 7. For the model including frailty, we ran the Monte Carlo chain for 23,000 iterations and discarded the first 3000 as burn-in. Just as for the simulations we initialised all frailties at the value one and took the initial value of $\xi$ to be 0.01. The trace plot in Fig. 2 shows how the estimate of $\xi$ quickly increased during the burn-in and then converged to a distribution centred just below 0.2. Trace plots for other parameters all also indicated convergence after burn-in. There are no maximised log likelihoods for the frailty model, given our use of MCMC estimation.

**Table 4:** Maximised log partial likelihood for no-frailty model

	Sale data	Scrap data
Null	$-$ 21597.01	$-$ 2331.58
No vessel effect $\beta _V=\theta _V$ = 0	$-$ 21287.56	$-$ 2256.48
No seller effect $\beta _S$ = 0	$-$ 21417.00	$-$ 2250.97
No exogenous effect $\beta _E=\theta _E$ = 0	$-$ 21298.04	$-$ 2287.95
No buyer effect $\beta _B=0$	$-$ 21353.07	NA
Full	$-$ 21262.85	$-$ 2235.24

**Table 5:** Vessel and company covariate effects for sales

	No frailty			Frailty		
	Est	SE	Wald	Est	SE	Wald
$$\beta _V$$						
Deadweight	− 0.010	0.004	− 2.48	− 0.006	0.004	− 1.44
Speed	− 0.053	0.019	− 2.75	− 0.048	0.020	− 2.35
Container	3.974	1.316	3.02	3.884	1.324	2.93
Previous owners	− 0.195	0.039	− 4.98	− 0.122	0.045	− 2.69
$$\beta _S$$						
Financial	0.437	0.129	3.39	0.263	0.165	1.59
Public	− 0.100	0.063	− 1.59	− 0.042	0.083	− 0.50
State	− 1.454	0.175	− 8.30	− 1.544	0.211	− 7.31
China	− 0.420	0.098	− 4.28	− 0.550	0.117	− 4.68
EMN	− 0.339	0.087	− 3.88	− 0.548	0.103	− 5.30
Germany	− 0.097	0.097	− 1.00	− 0.119	0.120	− 1.00
Greece	− 0.349	0.077	− 4.53	− 0.305	0.091	− 3.34
Japan	0.239	0.074	3.22	0.297	0.108	2.75
$$\beta _E$$						
Index	0.553	0.092	6.02	0.566	0.092	6.14
Lagged index	− 0.219	0.094	− 2.33	− 0.197	0.094	− 2.09
Index*Container	0.201	0.205	0.98	0.224	0.206	1.09
Lagged index*Container	− 0.639	0.212	− 3.01	− 0.649	0.212	− 3.05

**Table 6:** Company covariate effects for purchases

	No frailty			Frailty		
	Est	SE	Wald	Est	SE	Wald
$$\beta _B$$						
Financial	0.375	0.148	2.53	0.450	0.175	2.57
Public	0.098	0.073	1.33	0.114	0.092	1.24
State	− 0.048	0.133	− 0.36	− 0.114	0.172	− 0.66
China	− 0.098	0.087	− 1.12	− 0.171	0.107	− 1.59
EMN	− 0.275	0.077	− 3.55	− 0.270	0.095	− 2.86
Germany	− 0.655	0.111	− 5.92	− 0.641	0.130	− 4.94
Greece	0.256	0.063	4.09	0.206	0.078	2.63
Japan	− 1.124	0.137	− 8.22	− 1.086	0.159	− 6.82

**Table 7:** Vessel and company covariate effects for scrap

	No frailty			Frailty		
	Est	SE	Wald	Est	SE	Wald
$$\theta _V$$						
Deadweight	0.038	0.008	4.90	0.041	0.009	4.83
Speed	0.111	0.032	3.44	0.131	0.034	3.82
Container	7.977	4.642	1.72	8.607	4.675	1.84
Previous owners	− 0.138	0.054	− 2.56	− 0.063	0.060	− 1.06
$$\theta _S$$						
Financial	0.481	0.417	1.15	0.276	0.438	0.63
Public	− 0.136	0.136	− 1.00	− 0.306	0.154	− 1.98
State	0.216	0.201	1.08	0.003	0.240	0.01
China	0.277	0.166	1.67	0.362	0.188	1.93
EMN	− 0.126	0.169	− 0.75	− 0.283	0.183	− 1.55
Germany	0.533	0.192	2.78	0.486	0.229	2.12
Greece	0.239	0.152	1.57	0.265	0.167	1.59
Japan	0.861	0.204	4.21	1.045	0.242	4.31
$$\theta _E$$						
Index	− 0.726	0.279	− 2.60	− 0.697	0.279	− 2.49
Lagged index	− 0.992	0.265	− 3.74	− 1.007	0.266	− 3.79
Index*Container	− 0.413	0.500	− 0.82	− 0.441	0.502	− 0.88
Lagged index*Container	− 0.452	0.483	− 0.94	− 0.482	0.484	− 0.99

Conclusions concerning the regression coefficients are essentially the same whether or not frailty is included in the model. From Table 4 there are highly significant effects on sales intensity for covariates associated with the vessel and the buying and selling companies, and also relating to market conditions. The same is true for the intensity of vessel scrapping, except of course there is no buying company in that case.

The company-level effects are perhaps of most interest. State-owned companies are highly reluctant to sell, while Japanese and German companies are highly unlikely to buy vessels that are not new. Companies based in emerging maritime nations tend to either buy or sell relatively rarely. Greek companies, which are usually small and privately owned, are lively purchasers of second-hand vessels. The comments on Fig. 1 in Sect. 2 are consistent with these results. Interestingly, financial companies are likely to both sell and buy, which is in accord with suggestions that such companies are in a position to benefit from the volatile nature of the shipping industry due to availability of capital (Thanopoulou and Strandenes 2017).

Turning briefly to other effects, vessels are more likely to be traded when the Clarksea index is high, and more likely to be scrapped when it is low. The effect on containers is lagged compared with other vessel types. This may be because of the nature of the container sector where competition is linked to availability of capacity whose purpose is to provide efficient service to network strings. The container liner market is defined by high levels of cooperation and concentration (Sys 2009), which suggests that any capacity-related investment decisions need to be in alignment with the interests of strategic partners (Rau and Pinler 2016). If a liner operator believes that the cost of supply shortage is greater than the cost of excess capacity, they are likely to be optimistic when purchasing additional capacity (Fusillo 2003). Fusillo (2003) also provides evidence that: (1) capacity additions in the liner sector are large in comparison to demand and (2) that partially this is due to a strategic entry-deterring behaviour adopted by large operators where they use capacity expansion as means to limit competition. Therefore, the lagged effect might be a product of the resilience of liner operators due to cooperation and concentration of the market, the willingness to withstand market fluctuations rather than risk losing market share, the technical difficulties associated with rescheduling services and the time it takes to discuss capacity-related decisions with all strategic partners.

The mean of the frailty variance was ${\hat{\xi }}=0.231$ with MCMC standard deviation 0.027 after burn in. The distribution for individual company estimates ${\hat{Z}}_c$ was concentrated about one, as expected. Almost 60% of estimates were in the range (0.8, 1.2) and 94% in the range (0.5, 1.5). There were however some quite extreme values, with the smallest being 0.08 and the largest being 2.24. The smallest frailty estimate was for a large, state-owned Chinese company. Consistent with low frailty implying a preponderance of buying over selling, this company bought 25 vessels in our observation period and sold just one. At the other end of the scale the largest frailty estimate was for a medium sized privately owned Chinese company that sold eight vessels and bought none.

**Fig. 3:**
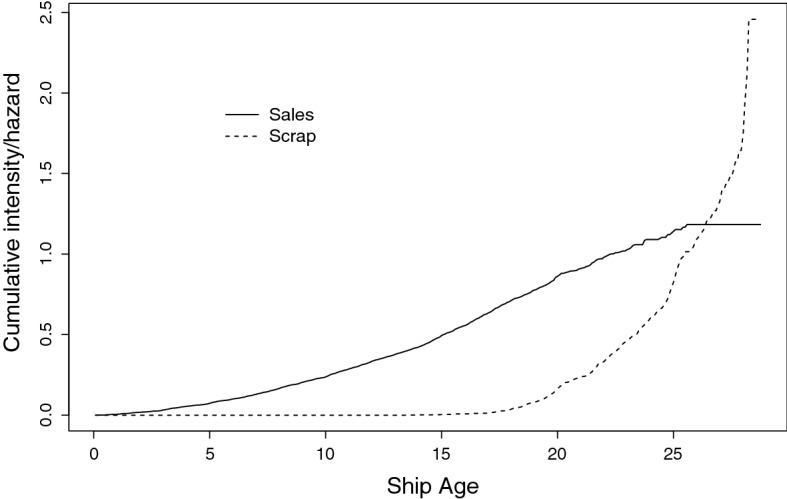
Estimated cumulative intensity for sales and cumulative hazard for scraps, evaluated at median values of covariates

Finally, Fig. 3 shows the effect of vessel age on intensity of sales or scrapping, evaluated for a vessel with median values of all covariates. Sales intensity is fairly constant until the vessel is around 25 years old, after which there are no sales. Vessels are not likely to be scrapped until they are around 20 years old, after which time the cumulative hazard for scrapping increases steeply year by year.

## Discussion

We have described a dual frailty model for ownership duration data and given an application on the buying, selling and scrapping of ocean-going vessels. The act of ordering new vessels was not considered due to the specific nature of newbuilding contracts in shipping. For example, the decision to order a vessel can be made years before the vessel is delivered to the buyer and, in addition, it is not uncommon for ships that have been ordered by one company to end up being delivered to another company. Subsequent purchases have a clearer process and have been, therefore, our focus in the paper.

**Fig. 4:**
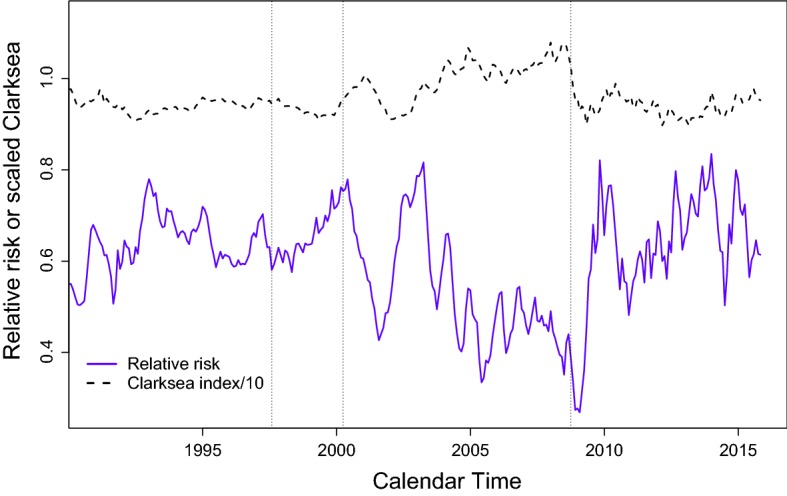
Relative risk of sale for container compared with other vessel types, together with logged Clarksea index (scaled by 10). The first vertical line marks the Asian financial crisis of July 1997, the second the peak in March 2000 of the dot.com bubble, and the third the financial crash of September 2008

We assumed the company-level frailty variables are time-constant and a natural extension would be to relax that assumption. A piecewise-constant model would seem to be appropriate, with change-points at some natural waymarks. In Fig. 4 for instance we show the logged Clarksea index together with the relative risk of sale for containers compared with other vessel types. We also indicate three events that could have serious effects on the market for vessels: the 1997 Asian financial crisis, the 2000 dot.com bubble and its demise, and the 2008 financial crash. After each of these there seems to be a change in slope for the relative risk. Allowing a company—if active—to have a different frailty for each of the periods between these events is a reasonable next step. Clearly the values are likely to be correlated, which could be accommodated in principle through the correlated gamma frailty models of Henderson and Shimakura (2003) or Fiocco et al. (2009). There is no simple form for the density of these multivariate gamma distributions and hence the use of MCMC would not be straightforward. Instead we might consider modelling the sentiments $S_c(t)$ directly, most obviously using a multivariate Gaussian distribution. In this case the price to be paid is the lack of a closed form conditional distribution, given the data and other frailties, which precludes the use of Gibbs sampling.

Another assumption is that the same sentiment or frailty affects buying, selling and scrapping. A potentially interesting extension would be to have three frailties per company, drawn from some trivariate distribution, with one acting on sales, one on purchases and one on scrapping. Another is to have a single frailty *Z* which acts directly on sales, say, but then to have powered forms $Z^{\gamma _1}$ and $Z^{\gamma _2}$ for the effects of sentiment on purchases and scrapping, respectively. A disadvantage of this model is that we would lose the generalised inverse gamma form for the posterior distributions of frailties. Further work on this would be worthwhile, and also for the time-varying situation. Clearly the available data would need to be rich in order for reliable inferences to be obtained from these more complex models.

## Electronic supplementary material

Below is the link to the electronic supplementary material.

Supplementary material 1 (pdf 179 KB)
