# Supplementary material for: A dual frailty model for lifetime analysis in maritime transportation
Source: Lifetime Data Anal. 2019 Feb 19;25(4):739–56. doi: 10.1007/s10985-019-09463-3 (PMC6776569; doi:10.1007/s10985-019-09463-3)
Supplement: Supplementary file 1 — Supplementary material 1 (pdf 179 KB) [file 10985_2019_9463_MOESM1_ESM.pdf]

A Dual Frailty Model for Lifetime Analysis in Maritime Transportation

Supplementary Material

In the simulation results reported in Section 4 we noted attenuation towards zero of the vessel and selling company regression estimates when frailty was ignored, but no apparent attenuation for buying company coefficients. We will briefly investigate.

For simplicity we assume no vessel or exogenous covariates and a single time-constant binary company covariate X^C. We use vessel age as the only time scale, in effect assuming that all vessels are delivered at the same calendar time. We assume that all companies are active throughout the observation period, that there is no scrapping and that all vessels are monitored, and can potentially be sold, up to age τ. Hence there are no calendar time effects and no selection effects due to vessels being observed or not. Interest is in the selling company and buying company regression coefficients β_S and β_B.

We consider three scenarios.

1. Sales are driven only by the selling company covariate and frailty. The transition intensity for a sale of vessel v at age a from its owning company $s(a, v)$ to another company b is

$$\alpha_v(a, b \mid \mathcal{F}_a, Z) = Z_{s(a,v)}\alpha_0(a)\exp\{\beta_S x^C_{s(a,v)}\}.$$

2. Sales are driven by the frailty and covariate of the buying company only. The intensity is

$$\alpha_v(a, b \mid \mathcal{F}_a, Z) = Z_b^{-1}\alpha_0(a)\exp\{\beta_B x^C_b\}.$$

3. Characteristics of both the selling and buying companies affect the sales intensity:

$$\alpha_v(a, b \mid \mathcal{F}_a, Z) = Z_{s(a,v)}Z_b^{-1}\alpha_0(a)\exp\{\beta_S x^C_{s(a,v)} + \beta_B x^C_b\}.$$

The simulation results in Table 1 support the suggestion in Section 4 that the coefficient β_S associated with the selling company is attenuated when

Table 1 Estimated regression coefficients under ignored frailty. Results based on 1000 replications with N=1000, $K = 1000$ and $\alpha_0(a)$=0.00001.

		$\xi = 0$		$\xi = 0.2$		$\xi = 0.4$	
Scenario	True	Mean	SE	Mean	SE	Mean	SE
1	β_S=1	1.000	0.029	0.933	0.047	0.860	0.058
	β_B=0	0.000	0.030	0.000	0.032	0.001	0.034
2	β_S=0	0.000	0.029	0.001	0.026	0.001	0.023
	β_B=1	1.002	0.031	1.002	0.045	1.004	0.083
3	β_S=1	1.000	0.023	0.923	0.063	0.774	0.162
	β_B=1	1.001	0.022	0.999	0.046	0.996	0.083

frailty is ignored, whereas the coefficient β_B associated with the buying company is not. In other simulations (not shown) we found the vessel-level regression coefficients are also attenuated under ignored frailty.

To explore a little, we consider estimation when both the number of vessels K and the number of companies N increase, not necessarily at same rate. Using similar methods to those of Henderson & Oman (1999), the estimators $\hat{\beta}_B$ and $\hat{\beta}_S$ can be shown to converge to so-called least-false values β_B^* and β_S^* respectively. These values are the solutions to the limiting score equations, with suitable scaling. We will concentrate first on Scenario 3, for which the limiting score equations are

$$0 = \int_0^\tau \Bigg\{ E_a\big[Z_{s(a)} Z_b^{-1} X_b^C \exp\{\beta_S X_{s(a)}^C + \beta_B X_b^C\}\big] - \frac{E_a[X_b^C \exp\{\beta_B^* X_b^C\}]}{E_a[\exp\{\beta_B^* X_b^C\}]} E_a\big[Z_{s(a)} Z_b^{-1} \exp\{\beta_S X_{s(a)}^C + \beta_B X_b^C\}\big] \Bigg\} \alpha_0(a) da \qquad (1)$$

for β_B, and

$$0 = \int_0^\tau \Bigg\{ E_a\big[Z_{s(a)} Z_b^{-1} X_{s(a)}^C \exp\{\beta_S X_{s(a)}^C + \beta_B X_b^C\}\big] - \frac{E_a[X_{s(a)}^C \exp\{\beta_S^* X_{s(a)}^C\}]}{E_a[\exp\{\beta_S^* X_{s(a)}^C\}]} E_a\big[Z_{s(a)} Z_b^{-1} \exp\{\beta_S X_{s(a)}^C + \beta_B X_b^C\}\big] \Bigg\} \alpha_0(a) da \qquad (2)$$

for β_S. Here $Z_{s(a)}$ and $X_{s(a)}^C$ are the frailty and covariate for the company owning a randomly chosen vessel at age a, and Z_b and X_b^C are the frailty and covariate for the buying company. The expectations are over the distributions of vessels and companies in place at age/time a.

First consider (1). Noting that the company distribution is time-constant in our scenarios and that the selling company is independent of the buying

company, we can simplify to

$$0 = \left\{ E\big[Z_b^{-1} X_b^C \exp\{\beta_B X_b^C\}\big] - \frac{E[X_b^C \exp\{\beta_B^* X_b^C\}]}{E[\exp\{\beta_B^* X_b^C\}]} E\big[Z_b^{-1} \exp\{\beta_B X_b^C\}\big] \right\}$$
$$\times \int_0^\tau E_a\big[Z_{s(a)} \exp\{\beta_S X_{s(a)}^C\}\big] \alpha_0(a) da.$$

Further, frailty Z_b is independent of X_b^C by assumption, and so (1) is equivalent to

$$0 = \left\{ E\big[X_b^C \exp\{\beta_B X_b^C\}\big] - \frac{E[X_b^C \exp\{\beta_B^* X_b^C\}]}{E[\exp\{\beta_B^* X_b^C\}]} E\big[\exp\{\beta_B X_b^C\}\big] \right\}$$
$$\times E\big[Z_b^{-1}\big] \int_0^\tau E_a\big[Z_{s(a)} \exp\{\beta_S X_{s(a)}^C\}\big] \alpha_0(a) da. \quad (3)$$

This is solved at $\beta_B^* = \beta_B$, the true value. Hence we expect consistent estimation of β_B in Scenario 3. Intuitively, the reason is that there is no selection effect that affects the buy-side of a transaction. All companies other than the seller are always potential buyers and their covariates and frailties are fixed. Hence we can factorise the limiting score as (3) and solve for β_B^* without considering the time-varying terms. The same can be shown for Scenario 2 and, for completeness, we can also show consistent estimation of $\beta_B = 0$ in Scenario 1.

The situation is different when we turn to estimating the coefficients β_S associated with selling. In (2), the terms involving the buying company are time-constant and independent of the selling company. Hence they can be factored out and the limiting score equation becomes

$$0 = \int_0^\tau \left\{ E_a\big[Z_{s(a)} X_{s(a)}^C \exp\{\beta_S X_{s(a)}^C\}\big] \right. \quad (4)$$
$$\left. - \frac{E_a[X_{s(a)}^C \exp\{\beta_S^* X_{s(a)}^C\}]}{E_a[\exp\{\beta_B^* X_{s(a)}^C\}]} E_a\big[Z_{s(a)} \exp\{\beta_S X_{s(a)}^C\}\big] \right\} \alpha_0(a) da.$$

Now the expectations involving the parameters of interest are not time-constant: we have a selection effect which does not apply to vessels directly, but rather to the companies owning vessels. Intuitively, as age a increases the distributions of $Z_{s(a)}$ and $\beta_S X_{s(a)}^C$ will become concentrated on values that are associated with low intensity of sales. As covariates are both observed and conditioned on, the change in distribution of covariates is correctly handled in the estimation process. The difficulty of course is that frailty is not observed and has been ignored in our estimation process. Attenuation might therefore be expected just as for standard survival analysis when frailty is ignored (Henderson & Oman 1999).

A difference is that for every sale there is a buyer in our transactions situation. Under our model we expect companies which have low intensity for sales to have high intensity for buying, after adjustment for covariates. Consequently we might expect the selection effects to stabilise once an equilibrium

Table 2 Estimates of β_S for Scenario 1 when frailty is ignored. The true value is β_S=1. Results are based on batches of 100 simulations, using frailty variance $\xi = 0.4$ and $\alpha_0(a) = 0.00001$.

		τ=300		τ=1000		τ=2000	
N	K	Mean	SE	Mean	SE	Mean	SE
1000	1000	0.860	0.059	0.903	0.059	0.940	0.056
10000	1000	0.865	0.040	0.912	0.029	0.941	0.026
1000	10000	0.950	0.066	0.981	0.071	0.995	0.084
10000	10000	0.951	0.019	0.978	0.021	0.990	0.025

is reached between the overall sales and buying intensities. To investigate, we will turn to Scenario 1, which has the same limiting score (4) as Scenario 3 but for which the equilibrium distribution is more easily obtained.

Let $p_{vc}(a)$ be the probability that vessel v is owned by company c at age a. In Scenario 1 there is no buyer effect and

$$p_{vc}(a+\delta a) = p_{vc}(a)\{1 - Z_c e^{\beta_S X_c^C}\alpha_0(a)\delta_a\} + \frac{1}{N-1}\left(\sum_{c'\neq c} p_{vc'}(a) Z_{c'} e^{\beta_S X_{c'}^C}\alpha_0(a)\delta_a\right) + o(\delta a).$$

Let π_{vc} be the equilibrium distribution. For global balance we must therefore have

$$\pi_{vc} \propto \frac{1}{Z_c e^{\beta_S X_c^C}} \qquad \sum_c \pi_v = 1$$

for all v and c.

Interestingly, if the expectations in (4) are determined at the equilibrium distribution then the integrand is zero when $\beta_S^* = \beta_S$. Hence we expect the least-false values to converge to the true values if τ is allowed to increase indefinitely. How much bias there is for fixed τ will depend on how quickly the equilibrium distribution is reached, which depends upon the parameter values, including N and K. Table 2 gives further simulation results to help explore. The bias reduces with increasing τ and K but not N, though increasing N reduces the standard error. Further analytic investigation may be worthwhile.

References

Henderson, R. & Oman, P. (1999), 'Effect of frailty on marginal regression estimates in survival analysis', *Journal of the Royal Statistical Society, Series B* **61**, 367–379.
